# Parliament and the Professions

**Published:** 1921-08-06

**Authors:** 


### Parliament and the Professions.

### Shell-shock Cases.

MR. MILLS asked the Minister of Pensions what is approximately the total number of pensioners now receiving treatment for war neurosis and other shell-shock conditions; and what is the number and proportion of these cases who have yet had no treatment or are awaiting treatment. Mr. Macpherson replied that there are approximately 3,400 men under treatment in institutions for disabilities of this nature. About 900 are awaiting admission, and, if unable to work, are receiving treatment allowances. In a large number of cases out-patient treatment is found to be sufficient.

### Hospital Grants and Wages.

MR. T. GRIFFITHS asked the Financial Secretary to the Treasury whether his attention had been drawn to a resolution passed by the Pharmacists' Branch of the National Drug and Chemical Union to the effect that no grants from public funds should be made to any hospital or other institution unless the provisions of the fair-wages clause are observed in the payment of employés; and whether he will give effect to this resolution and make the clause applicable in connection with grants to hospitals. Sir Alfred Mond said he had received a copy of the resolution in question, and would bring it to the notice of the Hospital Commission.

### Voluntary Hospitals.

MR. BRIANT asked the Minister of Health if, in the event of grants out of public funds being made to hospitals, provision will be made for representatives of the public to be on the governing bodies. Sir Alfred Mond said that he would bring the suggestion to the notice of the Hospitals Commission, but he doubted if a non-recurrent grant would justify a permanent change of this kind on the governing bodies being made.

### National Health Insurance.

MR. LYLE asked the Minister of Health whether about two million insured people entitled to receive medical benefits under the National Health Insurance Act did not appear at all on doctors' lists, from which inference is drawn that they pay their own doctors. Sir Alfred Mond replied that he thought that Mr. Lyle had been misinformed. There are always at any given moment certain insured persons who have not taken the trouble to select a doctor, but the number, of which no close estimate could be given, was but at any rate only a fraction of the two million in question. The inference to be drawn is not that such persons pay their own doctors, but that they too often delay in selecting a doctor until they require treatment.

